# Preparation of Hydrophobic Octadecylphosphonic Acid-Coated Magnetite Nanoparticles for the Demulsification of n-Hexane-in-Water Nanoemulsions

**DOI:** 10.3390/ma16155367

**Published:** 2023-07-31

**Authors:** Jiling Liang, Tingting Han, Wenwu Wang, Lunqiu Zhang, Yan Zhang

**Affiliations:** School of Civil Engineering, Liaoning Petrochemical University, Fushun 113001, China; l2j418@126.com (J.L.); 13478971740@163.com (T.H.); lxhwww520@163.com (W.W.); zhangyan_286192@163.com (Y.Z.)

**Keywords:** magnetite nanoparticle, O/W nanoemulsion, demulsification, octadecylphosphonic acid

## Abstract

To design more environmentally friendly, economical, and efficient demulsifiers for oily wastewater treatment, hydrophobic octadecylphosphonic acid (ODPA)-modified Fe_3_O_4_ nanoparticles (referred to as Fe_3_O_4_@ODPA) were prepared by condensation of hydroxyl groups between ODPA and Fe_3_O_4_ nanoparticles using the co-precipitation method. The prepared magnetite nanoparticles were characterized by X-ray diffraction (XRD), transmission electron microscopy (TEM), scanning electron microscope (SEM), Fourier transform infrared (FTIR) spectroscopy, and thermogravimetric/differential thermogravimetric (TG/DTG) analysis. The water contact angles (*θ*_W_) of Fe_3_O_4_@ODPA nanoparticles were more than 120°, indicating hydrophobic nature, and the diameter of the obtained spherical-shaped magnetite nanoparticles was 12–15 nm. The ODPA coating amount (*A*_O_) (coating weight per gram Fe_3_O_4_) and specific surface area (*S*_O_) of Fe_3_O_4_@ODPA were 0.124–0.144 g·g^−1^ and 78.65–91.01 m^2^·g^−1^, respectively. To evaluate the demulsification ability, stability, and reusability, the magnetite nanoparticles were used to demulsify an n-hexane-in-water nanoemulsion. The effects of the magnetite nanoparticle dosage (*C*_S_), pH value of nanoemulsion, and NaCl or CaCl_2_ electrolytes on the demulsification efficiency (*R*_O_) were investigated. The *R*_O_ of Fe_3_O_4_@ODPA samples was found to be higher than that of bare Fe_3_O_4_ samples (S0, ST, and SN) under all *C*_S_ values. With the increase in *C*_S_, the *R*_O_ of Fe_3_O_4_@ODPA samples initially increased and then approached equilibrium value at Cs = 80.0 g·L^−1^. A maximum *R*_O_ of ~93% was achieved at *C*_S_ = 100.0 g·L^−1^ for the Fe_3_O_4_@ODPA sample S2. The pH and two electrolytes had a minor effect on *R*_O._ The Fe_3_O_4_@ODPA nanoparticles maintained high *R*_O_ even after being reused for demulsification 11 times. This indicates that the hydrophobic Fe_3_O_4_@ODPA samples can be used as an effective magnetite demulsifer for oil-in-water nanoemulsions.

## 1. Introduction

At present, large amounts of oily wastewater are discharged from various industries, such as the petroleum [1,2,3,4,5], pesticide [6], pharmaceutical [7], and food [8] industries. Oily wastewater contains many organic pollutants and toxic chemicals, such as phenols, petroleum hydrocarbons, and polyaromatic hydrocarbons, which pose a serious threat to the environment and human health. Therefore, oily wastewater treatment has attracted considerable attention. Specifically, wastewater needs to be properly treated before being discharged into the external environment because it can increase the biochemical oxygen demand (BOD) and chemical oxygen demand (COD) in water resources. However, oily wastewater often contains interfacial-active materials, resulting in the formation of highly stable oil–water emulsions, which makes treatment quite difficult. Various effective treatment techniques have been developed for oily wastewater treatment [9,10,11,12,13,14,15,16,17,18,19], such as flotation and chemical coagulation [9,10,11,12,13], advanced oxidation processes [12,14,15], demulsification [16,17,18], membrane separation [17,19,20], freeze/thaw treatment [21,22], etc. Although these technologies are quite effective, they still have some limitations. For example, the chemical demulsification and chemical coagulation methods can generate secondary pollutants [19,23], the easy saturation of the membrane surface increases the operating cost of the membrane separation technique [24], and the operating cost and energy consumption of electrochemical separation methods are high due to the wastage of electrolytes and electrodes in the operating process [25]. Consequently, designing an environmentally friendly, economical, and efficient alternative demulsifier is imperative for oily wastewater treatment.

Recently, the magnetite oil–water separation technique has received extensive attention, as it offers rapid and efficient separation, recyclability, simple operation, low cost, and no secondary pollution [1,2,3,4,23,26,27,28,29,30]. Magnetite Fe_3_O_4_ nanoparticles, which are functionalized by organic and/or inorganic substrates, are used to demulsify oil-in-water or water-in-oil emulsions [1,2,3,26,27,31,32,33,34,35,36]. The functionalized magnetite nanoparticles can impart magnetite property to the dispersed oil droplets [3,4,23], which are coagulated rapidly and isolated from the continuous water phase under the application of an external magnetite field [3,4,23,29]. Since the magnetite nanoparticles can be continuously isolated, this separation technique is environmentally sustainable [2,3,4,26,29]. Various magnetite demulsifiers, such as magnetite amphiphilic composite [1], polyether polyol-modified magnetite [2], oleic acid-coated Fe_3_O_4_ nanoparticles [3], surface-active ethyl cellulose-grafted Fe_3_O_4_ nanoparticles [27], cyclodextrin-modified magnetite particles [29], magnetite nanoparticles grafted with amino groups [37] or tertiary amine polymer [38], and robust polymer-grafted Fe_3_O_4_ nanospheres [39], have been prepared and utilized for oil–water separation. However, more magnetite demulsifiers are needed to better understand their magnetite demulsification behavior.

**Figure 1:**
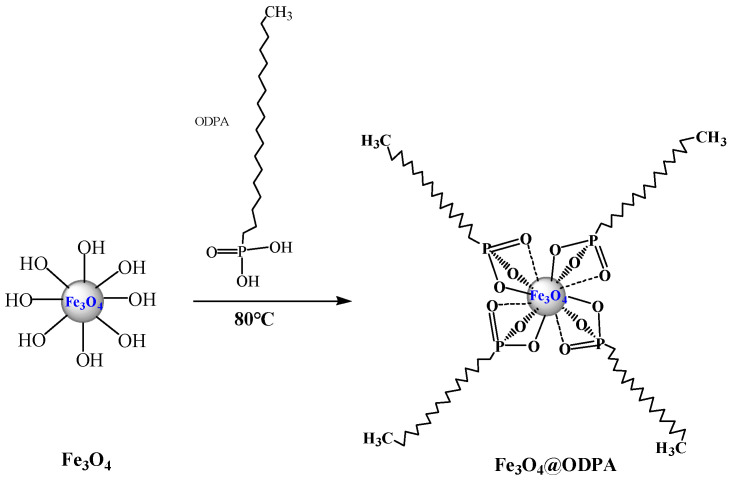
Schematic representing the interaction between ODPA and Fe_3_O_4_ surface.

Notably, hydrophobic octadecylphosphonic acid (ODPA) coatings were prepared on oxidized copper mesh for self-cleaning and oil/water separation and on titanium dioxide for anti-fouling biomedical applications [40,41]. In addition, ODPA coating has been used to modify the surface of Ti6Al4V alloys to improve the corrosion resistance [42]. The hydroxyl groups on the surface of TiO_2_ and Cu(OH)_2_ facilitate the coating of ODPA on the metal surface [40,41]. This provides a possibility to design a new magnetite demulsifier by using ODPA to modify Fe_3_O_4_ nanoparticles with ample hydroxyl groups under appropriate conditions. A possible schematic representing the interaction between ODPA and the Fe_3_O_4_ surface is shown in Figure 1. 

In this study, ODPA-modified Fe_3_O_4_ nanoparticles (Fe_3_O_4_@ODPA) are synthesized using co-precipitation with or without cetyltrimethylammonium bromide (CTAB). The prepared nanoparticles are characterized using X-ray diffraction (XRD), transmission electron microscopy (TEM), scanning electron microscopy (SEM), Fourier transform infrared (FTIR) spectroscopy, and thermogravimetric/differential thermogravimetric (TG/DTG) analysis. The demulsification ability and recyclability of Fe_3_O_4_@ODPA nanoparticles are investigated by using them to demulsify an n-hexane-in-water (O/W) nanoemulsion under an applied magnetite field. Furthermore, the effects of demulsifier dosage, pH, and electrolytes such as NaCl or CaCl_2_ on the removal efficiency (*R*_O_) of oil from the nanoemulsion are examined. 

## 2. Experiment

### 2.1. Materials

Analytical-grade ammonium hydroxide (25–28 wt% NH_3_ in water), n-hexane, ethanol, HCl, NaOH (granular), and oil-soluble Sudan III were purchased from Damao Chemical Reagent Factory, Tianjin, China. Analytical-grade reagents, including FeCl_3_∙6H_2_O, FeSO_4_∙7H_2_O, CTAB, NaCl, and CaCl_2_, and chemically pure Tween 60, were purchased from Sinopharm Chemical Reagent Co., Shanghai, China. Analytical-grade ODPA was purchased from Shanghai Bide Pharmatech, Shanghai, China. All of the reagents were directly used as received without further purification. The deionized water used in this study was obtained from a Hitech-Kflow water purification system from Hitech, Shenzhen, China.

### 2.2. Preparation of Fe_3_O_4_@ODPA Nanoparticles

A previously reported [3,43] modified chemical co-precipitation method was used to prepare the Fe_3_O_4_@ODPA nanoparticles. The preparation process of Fe_3_O_4_ nanoparticles can be found in our previous report [3]. Briefly, about 0.057 mol·L^−1^ of FeSO_4_∙7H_2_O and 0.123 mol·L^−1^ of FeCl_3_∙6H_2_O were dissolved in 350 mL deionized water under N_2_ flow. The above solution was heated to 80 °C under vigorous stirring. Next, 20 mL ammonium hydroxide was added to the solution. A black magnetite suspension (Fe_3_O_4_ nanoparticle suspension) was obtained, which was vigorously stirred for 30 min. Subsequently, nearly 0.50 g solid ODPA or alkaline ODPA solution (0.50 g ODPA dissolved in 50 mL alkaline solution) was added to the black magnetite suspension in the presence or absence of CTAB, and then the black suspension was continuously heated at 80 °C for 2 h. Next, the suspension was naturally cooled to room temperature, and the black magnetite nanoparticles were collected using a magnet, which were thoroughly washed with ethanol and deionized water in sequence. The obtained magnetite nanoparticles were dried in a vacuum oven at 60 °C to a constant weight, and Fe_3_O_4_@ODPA samples S1, S1T, and S2 were obtained. 

Bare Fe_3_O_4_ nanoparticles (S0, ST, and SN) were also synthesized for comparison. S0 and ST were prepared in the absence and presence of CTAB without adding ODPA using the same process as that for S1 and S1T, respectively. A bare Fe_3_O_4_ nanoparticle sample (SN) was prepared using the same process as that for S2 without adding ODPA. The diameter and water contact angle (*θ*_W_) of S0 were measured to be 11.3 nm and 29° at pH = 6.3 [3]. 

The preparation conditions and characterization of the prepared magnetite nanoparticles are shown in Table 1.

### 2.3. Preparation of n-Hexane-in-Water Nanoemulsions

**Figure 2:**
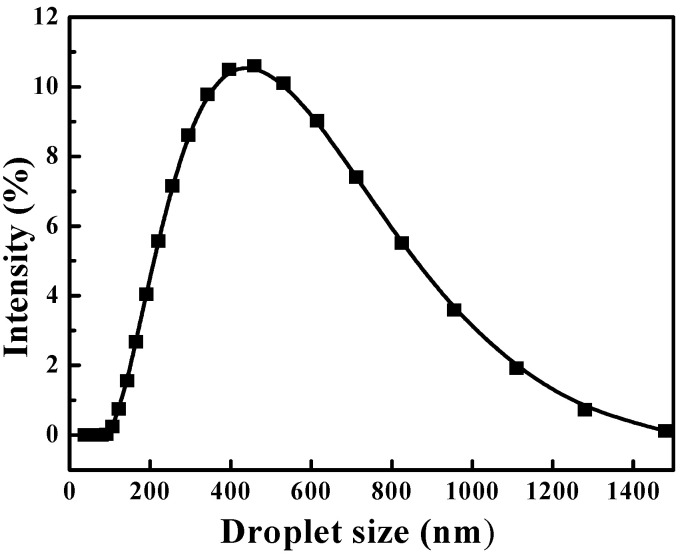
Droplet size distributions of nanoemulsion.

To obtain the parent n-hexane-in-water nanoemulsion, Tween 60, n-hexane (dyed using Sudan Ⅲ in oil phase), and deionized water were mixed in a mass ratio of 1:1:8 and stirred using a DS-1 homogenizer (Shanghai Specimen and Model Factory, Shanghai, China) at 10,000 rpm for 10 min. The parent nanoemulsion was diluted with an oil content of 25.0 g·L^−1^ and a mean droplet size of 311 nm (Figure 2), measured using a Zetasizer Nano S90 Mastersizer (Malvern Instruments Co., Malvern, UK), which was then used in the demulsification tests.

To investigate the effects of the pH value of the emulsion on the demulsification efficiency (*R*_O_), nanoemulsions were prepared using deionized water, whose pH value was previously adjusted by NaOH or HCl. To investigate the effects of NaCl or CaCl_2_ in the emulsion on *R*_O_, nanoemulsions were prepared using deionized water containing NaCl or CaCl_2_. The concentrations (*C*_Salt_) of NaCl or CaCl_2_ in deionized water were both 0.30 g·L^−1^.

### 2.4. Demulsification Tests of n-Hexane-in-Water Nanoemulsion 

To evaluate the demulsification ability of Fe_3_O_4_@ODPA for n-hexane-in-water nanoemulsion, the residual oil content (*C*_e_) of the nanoemulsion (light red transparent liquid) was measured after settling it on a hand magnet. The designed amount of magnetite nanoparticles was thoroughly mixed with 20 mL freshly prepared nanoemulsion in a 50 mL glass vial, where the dosage (*C*_S_) of magnetite nanoparticles was 0.0–100.0 g·L^−1^. The vials with the prepared mixtures were shaken in a THZ-82 thermostatic water bath shaker (Wuhan Grey Mo Lai Detection Equipment Co., Wuhan, China) under a speed of 240 cycles·min^−1^ at 25 °C for 3 h. The demulsification kinetic tests indicated that shaking for 3 h was enough to achieve demulsification equilibrium (seen Figure S1 in Supporting Information). Then, the magnetite nanoparticles and the adhered organics were separated by a 3000 Gs NdFeB magnet (Zibo Dry Magnetite Industry Science and Technology Co., Zibo, China). The absorbance was measured at 400 nm using a 754 ultraviolet-visible (UV-Vis) spectrometer (Shanghai Precision Scientific Instrument Co., Shanghai, China), which was then utilized to determine the residual oil content though a standard curve obtained from a series of standard nanoemulsions with different oil contents [4]. Finally, the demulsification efficiency (*R*_O_) could be calculated as follows:

*R*_O_ (%) = [(*C*_0_ − *C*_e_)/*C*_0_] × 100 (1)

where *C*_0_ (g·L^−1^) and *C*_e_ (g·L^−1^) are the initial and residual oil contents of the nanoemulsions, respectively.

The reported *R*_O_ values are the average values obtained from three parallel tests. For comparison, blank tests were conducted on nanoemulsions without the demulsifier. The relative error of the demulsification tests was <6.0%.

### 2.5. Recycling Tests

To evaluate the reusability and stability of Fe_3_O_4_@ODPA, Fe_3_O_4_@ODPA sample S2 was collected by an additional magnet after the demulsification test. Subsequently, it was sequentially washed with dichloromethane, ethanol, and water and then dried in a vacuum oven at 60 °C. This recycling test has been used in our reports [3,4]. The recycled Fe_3_O_4_@ODPA nanoparticles were applied in the subsequent demulsification tests 11 times to monitor any reduction in demulsification efficiency (*R*_O_).

### 2.6. Characterization of Magnetite Nanoparticles

The morphology of magnetite nanoparticles was characterized using TEM (JEM-F200 microscope, Jeol Corporation, Tokyo, Japan) and SEM (ZEISS sigma500, Zeiss, Jena, Germany). The crystal structures of the samples were examined by powder XRD (D8 advance model diffractometer, Bruker Co., Karlsruhe, Germany) with Cu *K*_α_ radiation (*λ* = 0.154184 nm) at 40 kV and 40 mA [3,4]. The chemical bonds in the samples were identified using FTIR spectroscopy (FTIR-660 + 610 spectrometer, Agilent Technologies Co., Palo Alto, USA) by the KBr wafers method. The ODPA coating mount on the Fe_3_O_4_ surface was measured by TG/DTG analysis (SDT-Q-600 thermal analysis system, TA Instruments Co., New Castle, DE, USA) [3]. In this analysis, the magnetite nanoparticles (~25 mg) were heated from room temperature to 800 °C at 10 °C·min^−1^ in an N_2_ atmosphere. The conventional drop shape method was used to measure the water contact angles (*θ*_W_) [3,44]. Firstly, a circular disc of magnetite nanoparticles with a thickness of approximately 2 mm was fabricated by a Shimadzu press at 10 MPa. Secondly, a droplet of deionized water (~3 μL) from a syringe was carefully dropped on the surface of the circular disc, and its image was taken after reaching equilibrium (~2 min) using a Datapyhsics OCA 20 contact angle goniometer (Kruss, Germany). The *θ*_W_ value was the average value measured at three different locations on the disc surface. 

The specific surface area (*S*_a_) of the magnetite nanoparticles was determined by N_2_ adsorption–desorption isotherm measurements (Quadrasorb SI-MP system, Quantachrome Instruments, Boynton Beac, Florida, USA), as shown in Table 1.

## 3. Results and Discussion

### 3.1. Characterization of Magnetite Nanoparticles

#### 3.1.1. Shape and Size of Magnetite Nanoparticles

**Figure 3:**
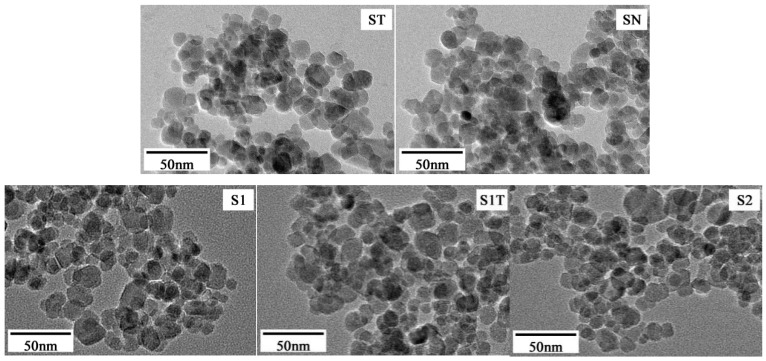
TEM images of magnetite nanoparticles.

**Figure 4:**
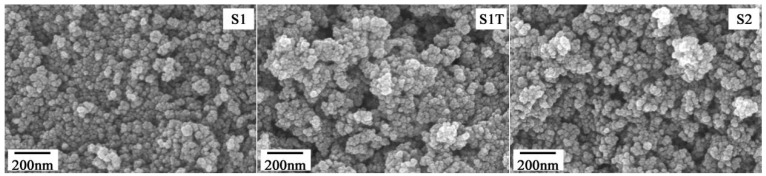
SEM images of magnetite nanoparticles.

The TEM and SEM images of magnetite nanoparticle shape are shown in Figure 3 and Figure 4, respectively. It is clear that all the magnetite nanoparticles had a spherical shape. Using the TEM images, the average diameter (*D*) of the prepared magnetite nanoparticles was measured to be 12.5–14.1 nm (see Table 1). 

#### 3.1.2. XRD Analysis

**Figure 5:**
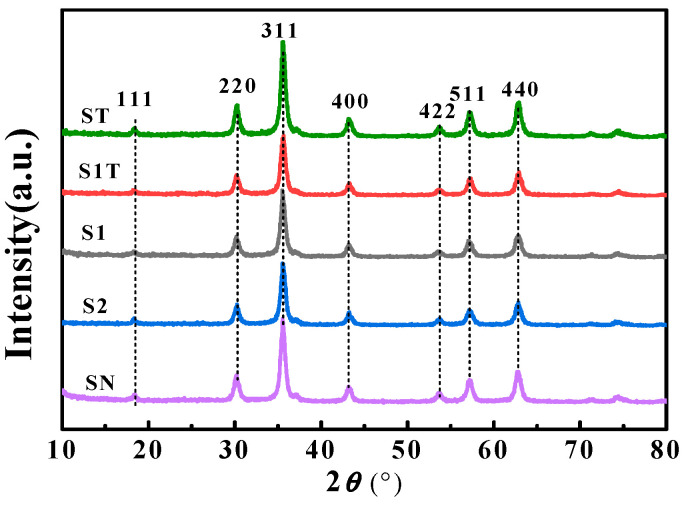
XRD patterns of magnetite nanoparticles.

The XRD patterns of the prepared magnetite nanoparticles are shown in Figure 5. All the samples exhibited peaks at 2*θ* of 18.4°, 30.4°, 35.5°, 43.3°, 53.7°, 57.4°, and 62.8°, which correspond to the (111), (220), (311), (400), (422), (511), and (440) diffraction planes, respectively, of the cubic spinel structure of Fe_3_O_4_ [3,33,34,45,46]. The XRD peak positions and intensities of Fe_3_O_4_@ODPA samples were similar to those of Fe_3_O_4_, indicating that the crystal structure of the magnetite particles were effectively retained after ODPA coating [2,3,44,45,47]. 

#### 3.1.3. FTIR Spectroscopy

**Figure 6:**
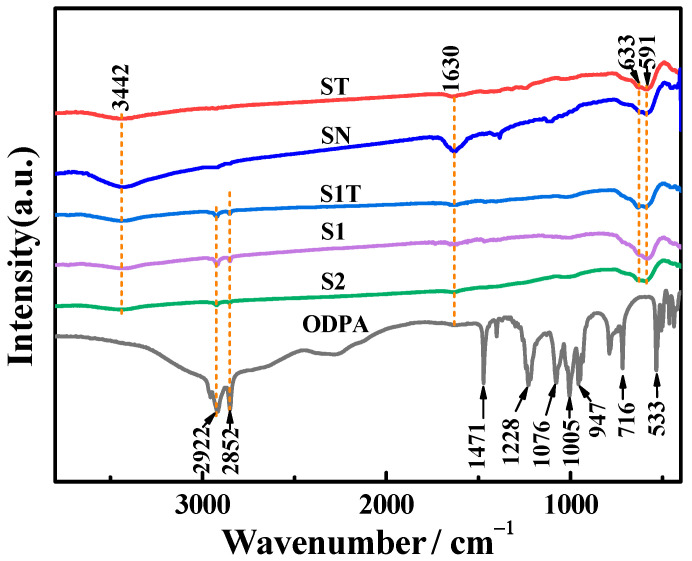
FT-IR spectra of magnetite nanoparticles and ODPA.

The FTIR spectra of solid ODPA and magnetite nanoparticles are shown in Figure 6. In the ODPA spectrum, the two sharp bands at 2922 and 2852 cm^−1^ were attributed to the characteristic asymmetric and symmetric C-H stretching vibrations, respectively [3,45,47,48]. The bands at 1471 cm^−1^ and 716 cm^−1^ were attributed to the *ν*(CH_2_) and *ν*(P-C) vibrations, respectively [49]. The absorption peaks at 1228 and 947 cm^−1^ were assigned to *ν*_as_(P=O) and *ν*_as_(P-OH), and the bands at 1076 and 1005 cm^−1^ were assigned to the symmetric *ν*_s_(PO_3_) and asymmetric *ν*_as_(PO_3_) stretching vibrations, respectively [40,50,51]. The spectra of bare ST and SN samples showed peaks at 3442 and 1630 cm^−1^, which were attributed to O-H vibrations. This indicates that abundant hydroxyl groups were present on the surface of the Fe_3_O_4_ nanoparticle, proving the rationality of the scheme shown in Figure 1. The bands at 630 and 591 cm^−1^ were assigned to Fe-O lattice vibrations [3,26,27,52,53]. The peaks at 2922 and 2852 cm^−1^, which correspond to Fe_3_O_4_ [3], were absent in the FTIR spectrum of ST, indicating that CTAB was not bonded with Fe_3_O_4_. 

The spectra of the Fe_3_O_4_@ODPA samples (S1, S1T, and S2) exhibited the characteristic ODPA bands at 2922 and 2852 cm^−1^ and bare ST and SN bands at 3442, 1630, 633, and 591 cm^−1^. The weakened peak intensities at 3442 and 1630 cm^−1^ in the Fe_3_O_4_@ODPA samples were attributed to the fact that some hydroxyl groups covering the surface were replaced by ODPA. ODPA can modify the Fe_3_O_4_ surface by monodentate, bidentate, or tridentate bonding [40,50]. The absence of peaks at 1228 and 947 cm^−1^ corresponding to ν_as_ (P=O) and ν_as_ (P-OH) vibrations indicates that ODPA was bound to the surface of Fe_3_O_4_ nanoparticles by tridentate bonding [51,54]. This inference is consistent with the design scheme shown in Figure 1. 

#### 3.1.4. TG/DTG Analysis 

**Figure 7:**
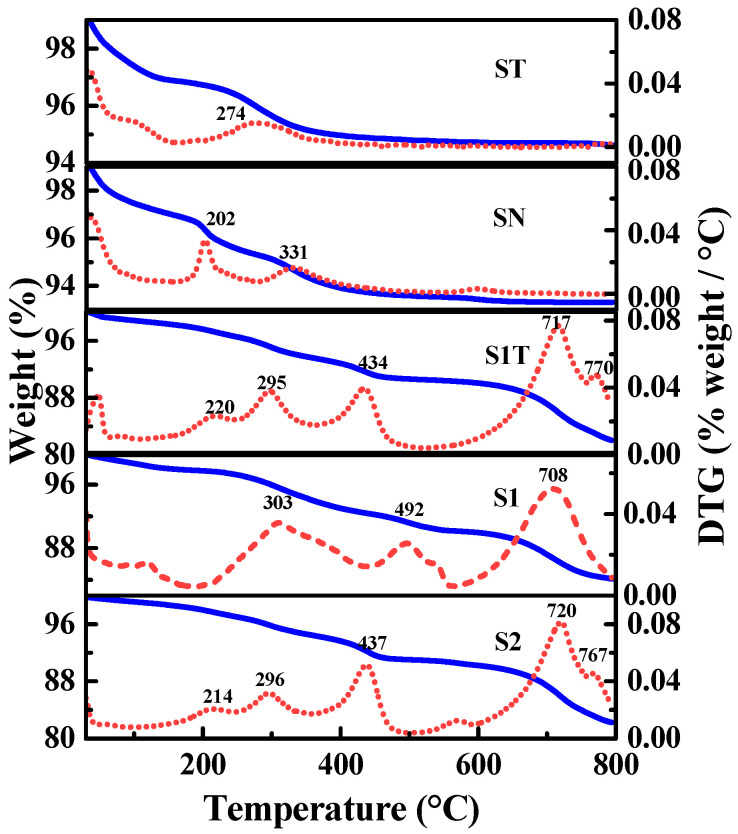
TG/DTG curves of bare magnetite nanoparticles. The red dash lines are the DTG data and the blue lines are TG data.

The TG/DTG curves of magnetite nanoparticles are shown in Figure 7. All the samples exhibited a weight loss peak below < 120 °C, which was ascribed to the desorption of physically adsorbed water. The bare Fe_3_O_4_ samples (ST and SN) showed a weight loss of 2.39 wt% and 3.84 wt% in the range of 120 °C to 600 °C, which was attributed to the condensation of surface hydroxyl groups [3,55]. The Fe_3_O_4_@ODPA samples (S1, S1T, and S2) exhibited two weight loss stages in the range of 120 °C to 800 °C. The first weight loss stage occurred at 120–600 °C due to the loss of the ODPA coated on the surface of magnetite nanoparticles [3,43,55,56]. In this stage, the weight loss occurred in two steps, as shown in the DTG curves in Figure 7. Two derivative peaks were observed at 202–331 °C and 437–492 °C, which were attributed to the breaking of different kinds of chemical bonds between -PO_3_^2−^ and Fe. The other weight loss stage appeared above 650 °C with a derivative peak at 708–770 °C, which may have been caused by the reduction of Fe_3_O_4_ by the reducing substances produced during the ODPA degradation process. This result is consistent with the existing literature [3,43,45,56,57].

The ODPA coating amount (*A*_O_) (g) on per gram Fe_3_O_4_ for the Fe_3_O_4_@ODPA samples was calculated using the net weight loss of ODPA from the TG data, and the results are shown in Table 1. The *A*_O_ values of S1 and S2 were 0.127 and 0.124 g·g^−1^. Thus, the average *A*_O_ value for S1 and S2 was approximately 0.126. However, the *A*_O_ value of S1T was 0.144 g·g^−1^, indicating that a slightly higher amount of ODPA was coated on the Fe_3_O_4_ surface when CTAB participated in the synthesis process.

### 3.2. Demulsification Performance of Magnetite Nanoparticles for Nanoemulsions 

#### 3.2.1. Effect of Demulsifier Dosage on the Demulsification Efficiency

**Figure 8:**
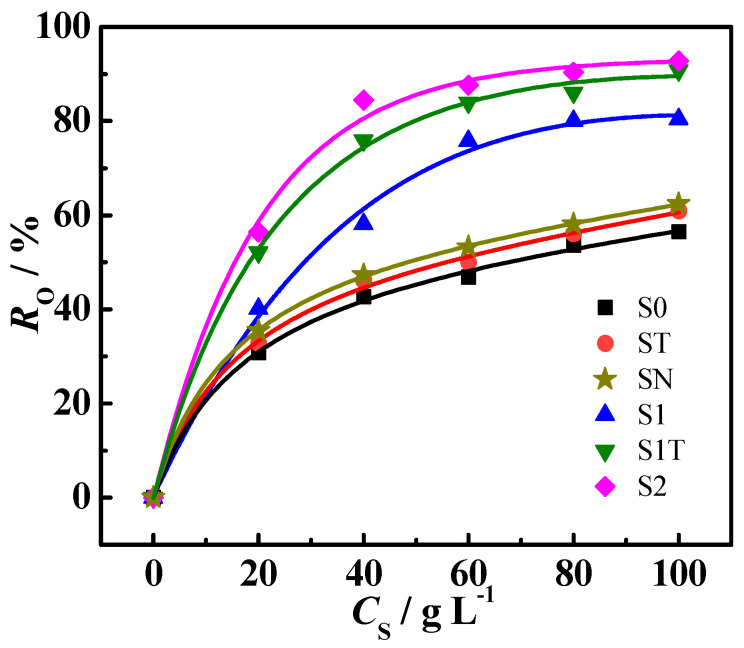
Effect of magnetite samples dosage (*C*_S_) on *R*_O_ for the nanoemulsion.

The magnetite nanoparticles were used as a demulsifier for n-hexane-in-water nanoemulsion. Figure 8 shows the influence of demulsifier dosage (*C*_S_) on the demulsification efficiency (*R*_O_) at PH = 6.9 and 25 °C. As *C*_S_ increased, the *R*_O_ of Fe_3_O_4_@ODPA nanoparticles (S1, S2, and S1T) initially increased and tended to stabilize at *C*_S_ ~80.0 g·L^−1^. The *R*_O_ values of all Fe_3_O_4_@ODPA samples were found to be higher than those of the bare magnetite samples (S0, ST, and SN). The order of *R*_O_ for the magnetite nanoparticles was S2 > S1T > S1 > bare Fe_3_O_4_ (SN, ST, and S0). The *R*_O_ values of the three bare Fe_3_O_4_ nanoparticles were very close to each other, nearly 60%, indicating that the bare Fe_3_O_4_ nanoparticles had some interfacial activity. A maximum *R*_O_ of ~93% was observed for the sample S2, verifying the potential of Fe_3_O_4_@ODPA nanoparticles in oil–water separation. 

The specific surface areas (*S*_a_) of determination were determined to further analyze the demulsification performance. The *S*_a_ values of Fe_3_O_4_@ODPA samples (S1, S1T, and S2) were 78.7, 87.0, and 87.0 m^2^·g^−1^, respectively, and less than those of the bare Fe_3_O_4_ samples (ST and SN) of 142.9 and 119.0 m^2^·g^−1^. The results do not seem to be related to demulsification performance. The relationship between specific surface area and demulsification ability needs to be studied further.

**Figure 9:**
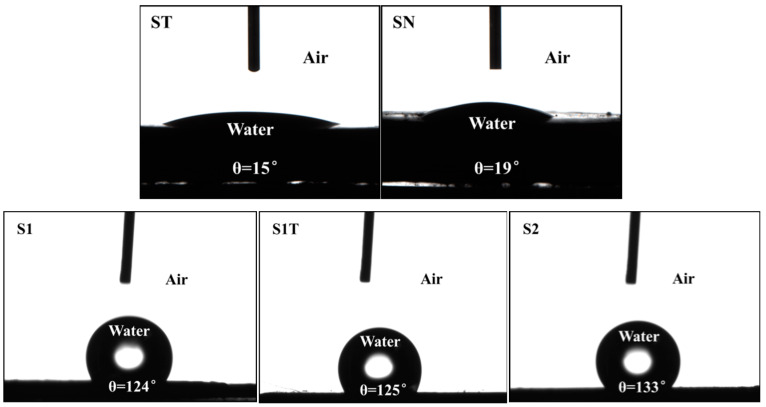
Representative water contact angles on the surface of magnetite nanoparticles.

It has been reported that the amount of coating modifier on the Fe_3_O_4_ surface and wettability can affect demulsification [3,58,59]. Therefore, the water contact angles of magnetite samples (pH 6.9) were also measured, and the results are shown in Figure 9. It can be seen that the *θ*_W_ values of the bare Fe_3_O_4_ samples (ST and SN) were 15° and 19°, respectively, indicating a hydrophilic nature. This is consistent with the hydrophilic Fe_3_O_4_ surface containing abundant hydroxyl groups. After coating ODPA, the interfacial activity of the magnetite nanoparticles increased, causing an increase in their demulsification ability. The *θ*_W_ values of Fe_3_O_4_@ODPA samples (S1, S1T, and S2) were 124°, 125°, and 133°, respectively, demonstrating that they were hydrophobic due to the ODPA coating. 

This suggests that samples S1, S1T, and S2 contained ODPA-coated structures, and the higher *θ*_W_ of S2 may have been the reason for its better demulsification performance. According to the above results, the bare Fe_3_O_4_ nanoparticles had a similar size, similar shape, approximately similar *θ*_W_ (<90°), and approximately similar demulsifying ability. This indicates that the properties of the bare Fe_3_O_4_ nanoparticles were unaffected by the addition of CTAB or alkaline aqueous solution in the absence of ODPA during the preparation process. 

#### 3.2.2. Effects of pH and Electrolytes on the Demulsification Efficiency

**Figure 10:**
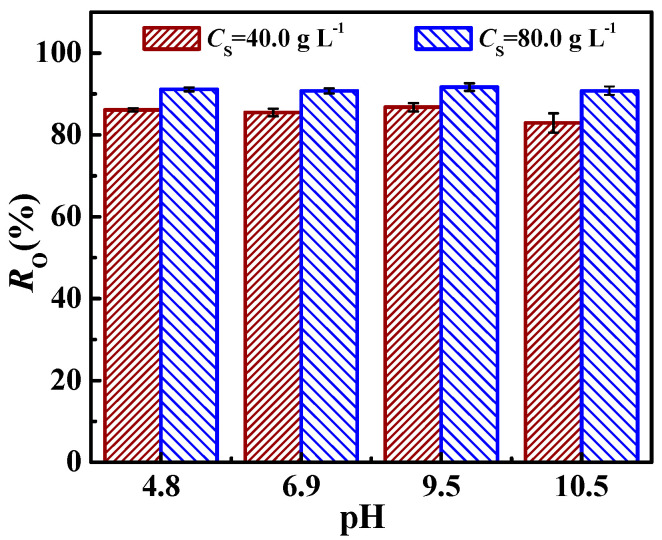
Influence of pH on *R*_O_ of S2 sample at *C*_S_ = 40.0 g·L^−1^ and 80.0 g·L^−1^.

**Figure 11:**
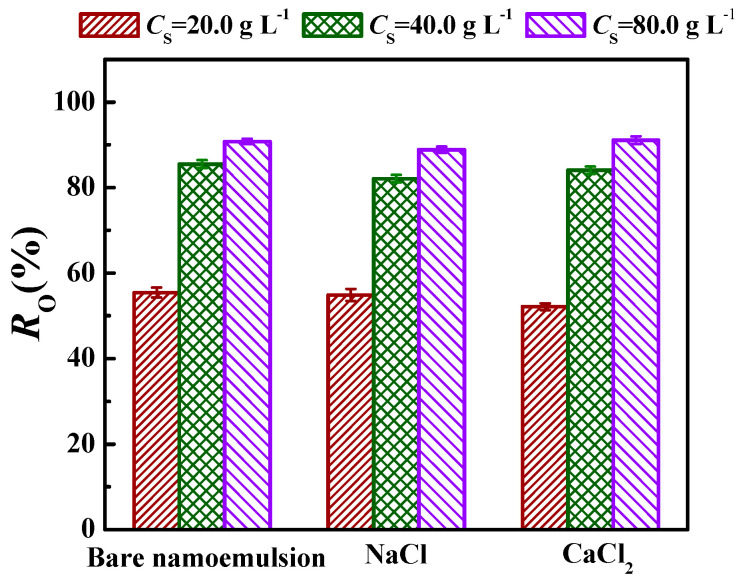
Influences of electrolytes (NaCl or CaCl_2_) on *R*_O_ of sample S2 at pH = 6.9 and *C*_Salt_ = 0.30 g·L^−1^.

The influences of pH and electrolytes (NaCl or CaCl_2_) on demulsification efficiency (*R*_O_) were investigated to assess the demulsifying property of Fe_3_O_4_@ODPA in different aqueous environments. Figure 10 shows the effect of the pH value of the nanoemulsion on the *R*_O_ of sample S2 under *C*_S_ = 40.0 g·L^−1^ and 80.0 g·L^−1^ at 25 °C. It can be seen that the *R*_O_ did not change significantly in the pH range of 4.8 to 10.5. This indicates that the pH value of nanoemulsion had a minor effect on the demulsification efficiency in the studied range. The result is consistent with an earlier report [3,34]. As was reported, the *R*_O_ of Fe_3_O_4_@PEI@β-CD and Fe_3_O_4_@OA was reduced from 96.8% to 95.3%, and there were no obvious differences as the pH of the emulsion increased from 4 to 10, respectively [3,34]. The effect of NaCl or CaCl_2_ with a concentration (*C*_salt_) of 0.30 g·L^−1^ on the *R*_O_ of sample S2 in the nanoemulsion at pH = 6.9 and 25 °C is shown in Figure 11. Compared with the n-hexane-in-water nanoemulsion without electrolyte (bare nanoemulsion), *R*_O_ changed slightly after adding a low concentration of NaCl or CaCl_2_ when *C*_S_ increased from 20.0 to 80.0 g·L^−1^. At *C*_S_ = 80.0 g·L^−1^, *R*_O_ was 90.8%, 88.9%, and 91.1% for the bare nanoemulsion, nanoemulsion containing NaCl, and nanoemulsion containing CaCl_2_, respectively. This is consistent with an earlier report about the demulsification of Fe_3_O_4_@OA for cyclohexane-in-water nanoemulsion [3].

Consequently, the variation in the pH value or the addition of low-concentration NaCl or CaCl_2_ (*C*_salt_ = 0.30 g·L^−1^) hardly affected the demulsification performance of Fe_3_O_4_@ODPA nanoparticles for n-hexane-in-water nanoemulsion.

#### 3.2.3. Recyclability Test of Fe_3_O_4_@ODPA

**Figure 12:**
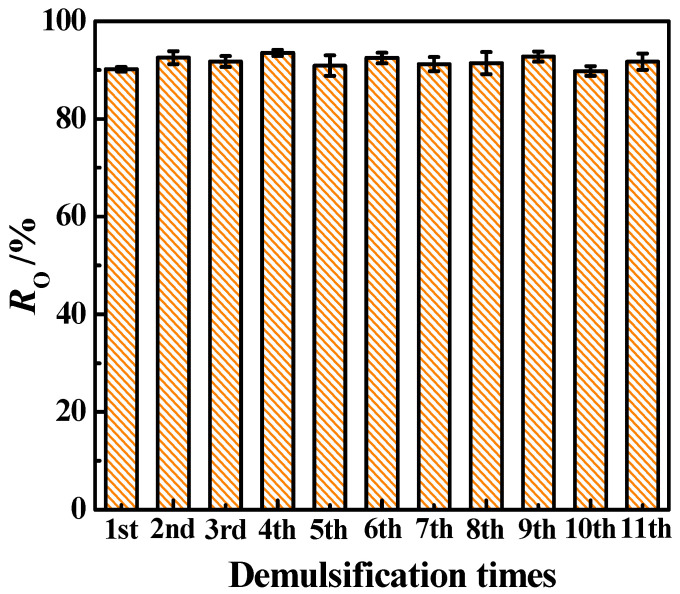
*R*_O_ of sample S2 during recycling tests at *C*_S_ = 80.0 g·L^−1^.

Compared with conventional chemical demulsifiers, magnetite demulsifiers can be recycled and reused to demulsify different nanoemulsions [2,3,26]. To evaluate the recyclability of hydrophobic Fe_3_O_4_@ODPA, the S2 sample was reused for demulsification 11 times. As shown in Figure 12, the sample still maintained a high *R*_O_ value after 10 cycles. Furthermore, the FTIR spectra and TG curves were obtained to observe the stability of the ODPA coating layer on the Fe_3_O_4_@ODPA surface (seen Figures S2 and S3 in Supporting Information), and no remarkable change was observed over the 10 cycles. This validates the good recycling performance of the hydrophilic Fe_3_O_4_@ODPA nanoparticles.

## 4. Conclusions

Hydrophobic and quasi-spherical Fe_3_O_4_@ODPA nanoparticles were prepared using the modified co-precipitation method. The size, *A*_O_, and *S*_O_ values of the Fe_3_O_4_@ODPA nanoparticles were approximately 12–15 nm, 0.124–0.144 g·g^−1^, and 78.65–91.01 m^2^·g^−1^, respectively. The *θ*_W_ of Fe_3_O_4_@ODPA nanoparticles was more than 120°, indicating hydrophobicity. The Fe_3_O_4_@ODPA nanoparticles exhibited efficient demulsification and excellent recyclability for n-hexane-in-water nanoemulsions under an external magnetite field. Further, the change in pH value of n-hexane-in-water nanoemulsion and the addition of a small amount of NaCl or CaCl_2_ in the nanoemulsion had a minor effect on the demulsification efficiency of Fe_3_O_4_@ODPA nanoparticles. Sample S2 exhibited a maximum *R*_O_ of 93% and excellent recyclability, with the highest *θ*_W_ of 133°. Overall, the findings of the study verify that Fe_3_O_4_@ODPA nanoparticles are an excellent demulsifier and can be effectively used for oily wastewater treatment.

## Figures and Tables

**Table 1:** Preparation conditions and characterization of the magnetite nanoparticles.

Sample	Preparation Conditions			*D* (nm)	*S*_a_ (m^2^·g^−1^)	*θ*_W_ (°)	*A*_O_ (g·g^−1^)
	CTAB	Alkaline Solution	*A*_D_ (g)				
ST	Presence	Absence	-	12.0	142.9	15	-
SN	Absence	Presence	-	11.2	119.0	19	-
S1	Absence	Absence	0.50	14.1	87.0	124	0.13
S2	Absence	Presence	0.50	12.5	87.0	133	0.12
S1T	Presence	Absence	0.50	13.9	78.7	125	0.14

*A*_D_ in Table 1 is the amount of ODPA added in the preparation process of Fe_3_O_4_@ODPA nanoparticles.

## Supplementary Materials

The following supporting information can be downloaded at: https://www.mdpi.com/article/10.3390/ma16155367/s1.
