# Supplementary material for: Preparation of Hydrophobic Octadecylphosphonic Acid-Coated Magnetite Nanoparticles for the Demulsification of n-Hexane-in-Water Nanoemulsions
Source: Materials (Basel). 2023 Jul 31;16(15):5367. doi: 10.3390/ma16155367 (PMC10419870; doi:10.3390/ma16155367)
Supplement: Supplementary file 1 [file materials-16-05367-s001.zip › materials-2519589-supplementary.pdf]

Supporting Information

Preparation of Hydrophobic Octadecylphosphonic Acid-Coated Magnetite Nanoparticles for the Demulsification of n-Hexane-in-Water Nanoemulsions

Jiling Liang, Tingting Han, Wenwu Wang, Lunqiu Zhang *, Yan Zhang

School of Civil Engineering, Liaoning Petrochemical University, Fushun 113001, China; l2j418@126.com (J.L.); 13478971740@163.com (T.H.); lxhwww520@163.com (W.W.); zhangyan_286192@163.com (Y.Z.)

* To whom correspondence should be addressed

Tel.: +86 186-4011-9142

E-mail: zhangdoctor2022@126.com

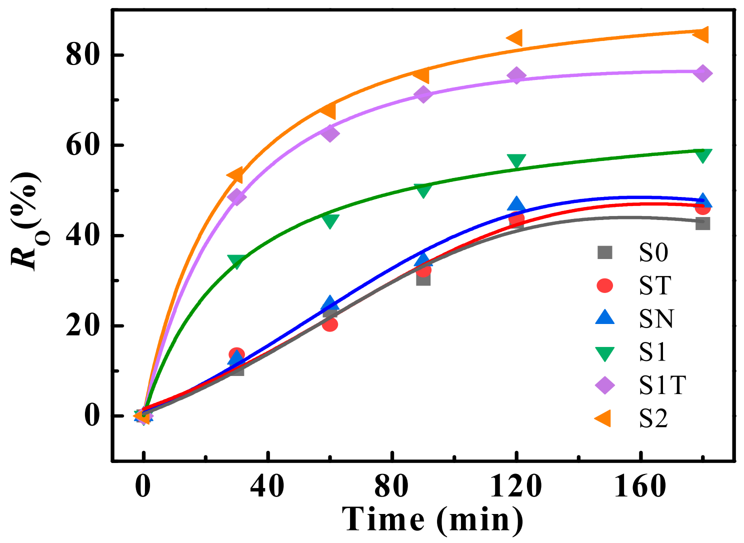

Figure S1 Plots of demulsificartion efficiency (R_O) *vs*. time at C_S = 40.0 g·L^{-1}.

† The effect of time on demulsification efficiency (R_O) for magnetic nanoparticles was determined by shaking 0-180 min at C_S=40.0 g L^{-1}. The results show that the R_O tended to reach an equilibrium value after demulsifying for 120 min. It is indicated that shaking 3 h is sufficient to reach demudification equilibrium.

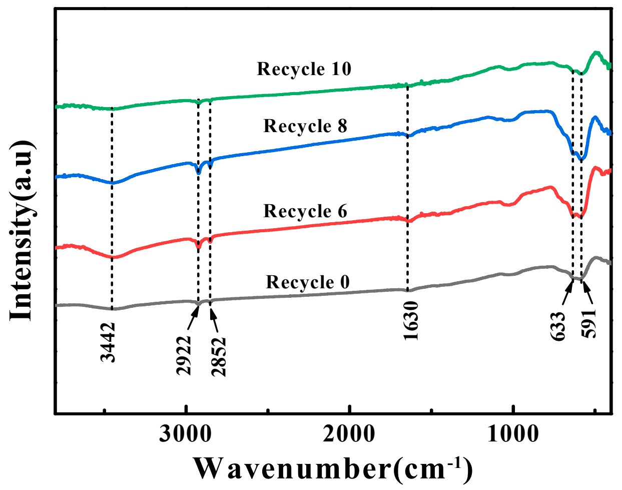

Figure S2 FT-IR spectra of S2 sample recovered in Recycling tests

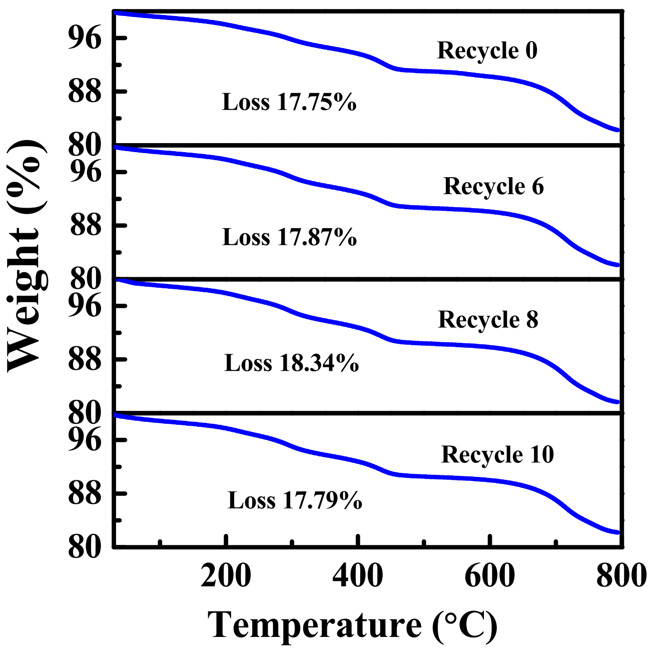

Figure S3 TG curves of S2 sample recovered in Recycling tests
